# Effect of Early Age-Curing Methods on Drying Shrinkage of Alkali-Activated Slag Concrete

**DOI:** 10.3390/ma12101633

**Published:** 2019-05-18

**Authors:** Yuxin Cai, Linwen Yu, Yong Yang, Yang Gao, Changhui Yang

**Affiliations:** 1 College of Materials Science and Engineering, Chongqing University, Chongqing 400045, China; yuxin.cai@cqu.edu.cn (Y.C.); y.yong@cqu.edu.cn (Y.Y.); yang.gao@cqu.edu.cn (Y.G.); 2 State Key Laboratory of Green Building Materials, Beijing 100024, China; 3 Chongqing Engineering Research Centre for High Performance Concrete, Chongqing 400045, China

**Keywords:** alkali-activated slag concrete (AASC), curing, drying shrinkage, pore structure

## Abstract

Drying shrinkage of alkali-activated slag concrete (AASC) is significantly greater than that of concrete made with ordinary Portland cement (OPC). It limits the large-scale application of AASC in field engineering. This study investigates the effect of early age-curing methods, including water curing, curing in elevated-temperature water, and CO_2_ curing, on drying shrinkage of AASC. Scanning electron microscopy (SEM), X-ray diffraction (XRD), thermogravimetric (TG-DTG), and mercury intrusion porosimetry (MIP) were carried out to analyze the composition and microstructure of hydration products, to provide deeper understanding of drying shrinkage of AASC. The results show that water curing decreased drying shrinkage of both C30 and C50 AASC moderately compared to air curing, while it was more effective for C30 AASC. Curing in water of elevated temperature and CO_2_ curing were very beneficial to mitigate drying shrinkage of AASC. Heat curing decreased drying shrinkage of AASC up to 80%. SEM and TG-DTG results show that denser microstructure formed because of the accelerated hydration, resulting in lower porosity and lower proportion of pores smaller than 25 nm that contributed to the reduction of drying shrinkage. In addition, under high-temperature curing, most autogenous shrinkage of AASC occurred in the first few days because hydration was accelerated. After measurement of drying shrinkage was started, recorded autogenous shrinkage of AASC cured in elevated-temperature water should be much less than that of AASC cured at normal temperature. It is another important reason for the reduction of drying shrinkage. Carbonation occurring in the CO_2_ curing period led to the decalcification of C-(A)-S-H gel; it coarsened the pore-size distribution and decreased the total porosity. Therefore, drying shrinkage of C30 and C50 AASC was declined by 49% and 53% respectively.

## 1. Introduction

Alkali-activated slag (AAS) cement is a novel type of clinker-free binder material prepared by blast furnace slag and alkaline solution, such as sodium hydroxide and water glass. The embodied energy and CO_2_ emission could be reduced significantly due to the use of AAS as an alternative to ordinary Portland cement (OPC) [1,2,3,4,5], therefore much attention has been paid to the properties of AAS in recent decades. Compared to OPC, AAS cement exhibits lower hydration heat and faster hydration rate [6,7,8]. Furthermore, alkali-activated slag concrete (AASC) has high early strength, good resistance to permeability, chemical attack, and freeze-thaw cycles. It is a green building material of energy-saving, waste-eliminating and environmentally friendly [9,10,11,12].

However, it is widely reported that drying shrinkage of mortars and concretes made with AAS cement was considerably higher than that of OPC. Atişe et al. [13] found that drying shrinkage of sodium silicate activated slag mortar was 3 to 6 times that of OPC mortar. The research of Collins and Sanjayan [14,15] show that under the same curing conditions, drying shrinkage of AASC was about 2 to 3 times that of OPC concrete. It is well known that surface tension, capillary stress and disjoining pressure were the main mechanisms for drying shrinkage of cementitious materials [16,17]. Cartwright et al. [18] and Lee et al. [19] attributed the higher drying shrinkage of AASC than OPC concrete to the greater pore capillary stress of AASC, which was induced by the finer pore size. Ye et al. [20,21] believed that the large shrinkage in AASC might be caused by the structural incorporation of alkali cations in C-(A)-S-H, which reduced the stacking regularity of C-(A)-S-H gel layers and made the C-(A)-S-H easier to collapse and redistribute upon drying.

The higher shrinkage deformation will seriously affect the bearing capacity and durability of concrete structures, then limit the large-scale application of AASC in practical engineering. Therefore, how to decrease the shrinkage deformation of AASC has become a big problem to be solved urgently. Previous studies [22,23,24] showed that shrinkage of concrete was closely related to their early age curing. Thomas et al. [16], Bilim et al. [25] and Bakharev et al. [26] found that heat curing (curing under elevated temperature) accelerated strength development obviously and reduced drying shrinkage of AASC significantly. The considerable decrease in porosity, water loss and the increase in stiffness of microstructure were considered to be the main reasons for the restrain of drying shrinkage [16]. Water curing in the early age was also found to reduce drying shrinkage of AASC effectively, because the AASC specimens were denser and more homogeneous [27]. In recent years, CO_2_ curing concrete technology has attracted wide attention [28,29,30]. The published results show drying shrinkage of concrete cured in CO_2_ significantly decreased due to the reduction of water loss rate [31]. It should be noted that the alkalinity of concrete decreased due to the application of CO_2_ curing, so steel reinforcements in the CO_2_ cured concrete became vulnerable to corrosion. Until now CO_2_ curing has been carried out only on concrete made with OPC, little attention has been paid on the properties of AASC cured in CO_2_.

Those studies mentioned above have important reference significance on how to reduce drying shrinkage of AASC; however, the working mechanism of different curing methods on mitigating drying shrinkage deformation of AASC needs further investigation. In this paper, drying shrinkage and water loss rate of concrete cured under different conditions were studied. The curing temperature, relative humidity (RH) and CO_2_ curing were considered in the present investigation. Meanwhile, hydration products and pore structures of AAS pastes under different curing conditions were also studied to provide deeper understanding of drying shrinkage of AASC.

## 2. Materials and Methods

### 2.1. Raw Materials and Mixture Proportion

**Figure 1:**
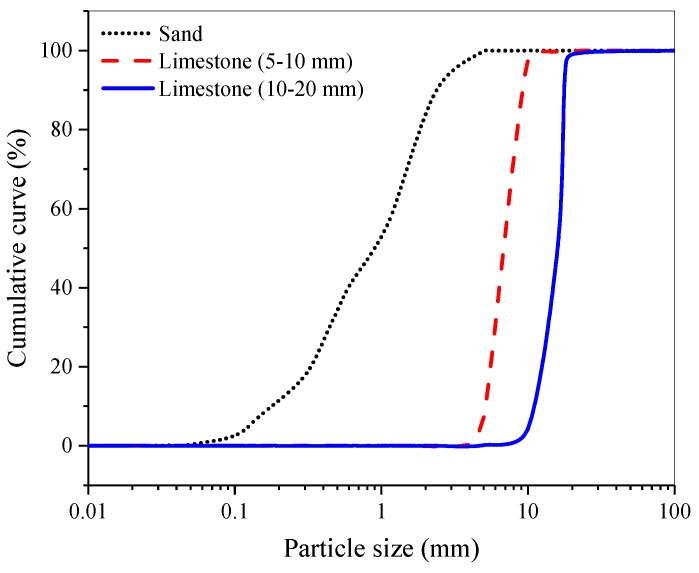
Particles size distribution of sand and crushed limestone.

The used ground granulated blast furnace slag (GGBFS) was taken from Chongqing Iron and Steel Group Co., Ltd., its density was 2.91 g/cm^3^ and Blaine specific surface area was 420 m^2^/kg. The main chemical compositions of GGBFS are shown in Table 1. Water glass and sodium hydroxide were used as activator. Water glass used was an industrial grade sodium silicate, the main physical properties and chemical compositions of water glass are shown in Table 2. Sodium hydroxide in pellet form was an industrial grade with a purity of 99%. The alkaline activator consisting of water glass and sodium hydroxide with a molar modulus of 1.5 was prepared 24 h prior to use. Manufactured sand was selected as the fine aggregate, the fineness modulus was 2.9, other physical properties are shown in Table 3. Crushed limestone with particle sizes of 5–10 mm and 10–20 mm was used as coarse aggregate, mixed with a mass ratio of 4:6. The main chemical compositions of crushed limestone are shown in Table 4 and the basic performance of crushed limestone is shown in Table 5. Particles size distribution of sand and limestone is presented in Figure 1.

Two concretes, with a target compressive strength grade of C30 and C50 respectively, were studied in this paper. The mixture proportion of AASC is shown in Table 6. Both had a binder (GGBFS) content of 400 kg/m^3^ and the alkali content (the dosage of sodium oxide in percent by mass of binder) of 5%. Water to binder ratio (w/b) was adjusted to achieve the target compressive strength.

### 2.2. Test Methods

#### 2.2.1. Early Age Curing

The purpose of this study is to investigate the effects of early age-curing temperature, RH, and CO_2_ curing on drying shrinkage of AASC. Seven different early age-curing regimes, including water curing at three different temperatures, two air curing at different RH, and two CO_2_ curing at different RH, were designed. The detailed information of each curing regime is presented in Table 7. Water curing at the temperatures of 20 °C, 40 °C and 60 °C was carried out in a temperature-controlled tank, and the AASC specimens were immersed under water during the whole curing period. The air curing at different RH was carried out in a humidity-controlled chamber. Some AASC specimens were put into a CO_2_ curing chamber to apply CO_2_ curing. The concentration of CO_2_ was (20 ± 3)% and the pressure was standard atmospheric pressure.

#### 2.2.2. Compressive Strength

Cubic specimens with a dimension of 100 mm × 100 mm × 100 mm were prepared for compressive strength test. Cast specimens were sealed with a plastic film to prevent moisture loss and then stored at (20 ± 2) °C and >90% RH for 24 h. The specimens were demolded after 24 h and then cured in a standard curing room with a temperature of (20 ± 2) °C and RH of more than 95%. Referring to Chinese standard GB/T50081-2002 "Standard for Test Methods of Mechanical Properties of Concrete", the compressive strength was measured at 3 d, 7 d, and 28 d respectively, the applied loading rate was 0.5 MPa/s and each result was obtained from three duplicated specimens.

#### 2.2.3. Drying Shrinkage and Moisture Loss

Prismatic specimens with a size of 100 mm × 100 mm × 515 mm were prepared for drying shrinkage test, while cubic specimens with a size of 100 mm × 100 mm × 100 mm were prepared for moisture loss test. The specimens were demolded after 24 h curing at (20 ± 2) °C and >90% RH, then subjected to seven different early age-curing methods. After the curing was completed, the specimens were removed into a drying shrinkage chamber where the temperature was controlled at (20 ± 2) °C and the RH was controlled at (60 ± 5)%. Residual water on the samples cured in water was removed with a wet towel. Two hours later, the initial length of prismatic specimens and the initial weight of cubic specimens was recorded. Drying shrinkage and moisture loss of specimens were measured at 1 d, 3 d, 7 d, 14 d, 21 d, 28 d, 56 d, and 90 d, and each result was obtained from three duplicated specimens.

#### 2.2.4. Other Tests

To get deeper understanding on drying shrinkage of AASC cured in different conditions, scanning electron microscopy (SEM) manufactured by Tescan in the Czech Republic, X-ray diffraction (XRD) manufactured by Rigaku in Japan, thermogravimetric (TG-DTG) manufactured by Netzsch in Germany, and mercury intrusion porosimetry (MIP) manufactured by Micromeritics in the United States were carried out to analyze the composition and microstructure of hydration products. However, the random distribution of aggregate in concrete led to high variable results of these test. Therefore, all the results of SEM, XRD, TG-DTG, and MIP reported in this paper were based on paste samples. AAS pastes were prepared at a constant w/b of 0.42 and alkali dosage of 5%. The modulus of alkali activator was 1.5. After 24 h sealed curing at (20 ± 2) °C and >90% RH, the samples were demolded. Then they were exposed to seven different curing regimes for 48 h. Finally, the samples were cut into pieces of about 5 mm, and hydration was terminated with anhydrous ethanol. Before the test, the samples were placed in a vacuum drying oven at 60 °C to a constant weight for use.

The microstructure of AAS pastes was observed with a VEGA3 LMH tungsten filament SEM at an accelerating voltage of 14 kV. The MIP test was conducted on a Micrometrics Autopore Mercury Porosimeter IV 9500 with a pressure range of 0.05 to 50,000 Psi pressure. XRD was carried out on a D/Max-5A12kW target X-ray diffractometer with CuKα radiation. The powder samples passing through 160 μm sieve were step-scanned from 5 to 70° (2θ) at a rate of 4°/min. TG-DTG test was tested with a STA-449C TG-DSC integrated thermal analyzer. The heating range was (20 ± 1) °C to 1000 °C, the heating rate was 20 °C/min and the heating atmosphere was argon.

## 3. Experimental Results

### 3.1. Compressive Strength of AASC Cured under Different Conditions

**Figure 2:**
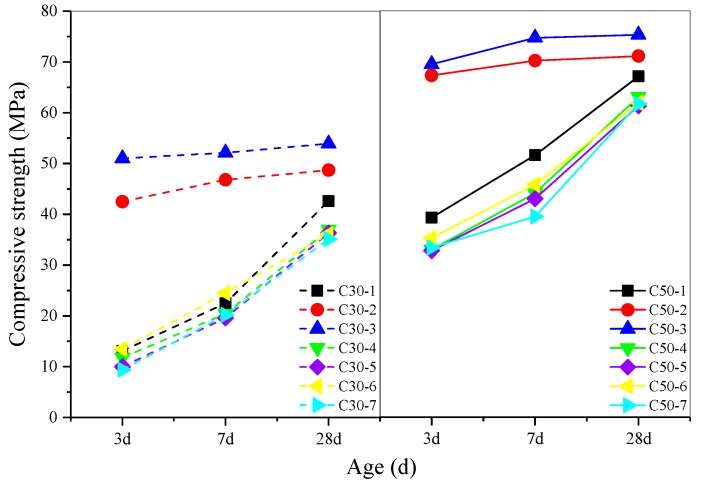
Effect of early age curing on compressive strength of AASC.

The results of compressive strength measurements of AASC cured under different conditions are presented in Figure 2. For the specimens cured under the same curing condition, the compressive strength of AASC at lower w/b was always significantly higher. It is quite logical because higher w/b results in higher porosity and looser microstructure. When cured in high-temperature water, compressive strength of AASC developed quite rapidly. The 3-d compressive strength of concrete cured in 40 °C water exceeded the 28-d compressive strength of concrete cured in 20 °C water, but higher temperature resulted in slower strength development in the later age. This is consistent with previous published results [16,25,26,32]. Furthermore, higher RH in the early age-curing period helped AASC to achieve higher compressive strength. Because sufficient water in high RH environment was conducive to the early hydration reaction of the slag, resulting in more hydration products and a tighter concrete structure [14,15,27,33]. However, it can be found that the influence of RH on compressive strength of AASC is not so important as the effect of curing temperature.

The results in Figure 2 show that CO_2_ curing did not affect compressive strength of AASC obviously. Because in CO_2_ curing environment, hydration products of AASC reacted with CO_2_ to form CaCO_3_ which was extremely insoluble in water. On the one hand, the formation of CaCO_3_ precipitated in the pores of concrete and made the concrete denser. This is positive for compressive strength development of AASC [34,35]. On the other hand, carbonation caused the decalcification of C-(A)-S-H gel, which resulted in partial strength loss [28,36]. Therefore, considering these two aspects, CO_2_ curing had little effect on compressive strength of AASC.

### 3.2. Drying Shrinkage and Moisture Loss of AASC Cured under Different Conditions

**Figure 3:**
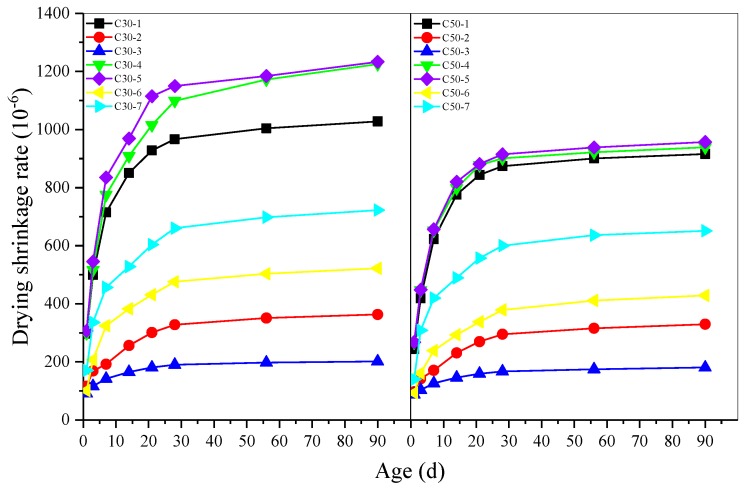
Effect of early age curing on drying shrinkage of AASC.

**Figure 4:**
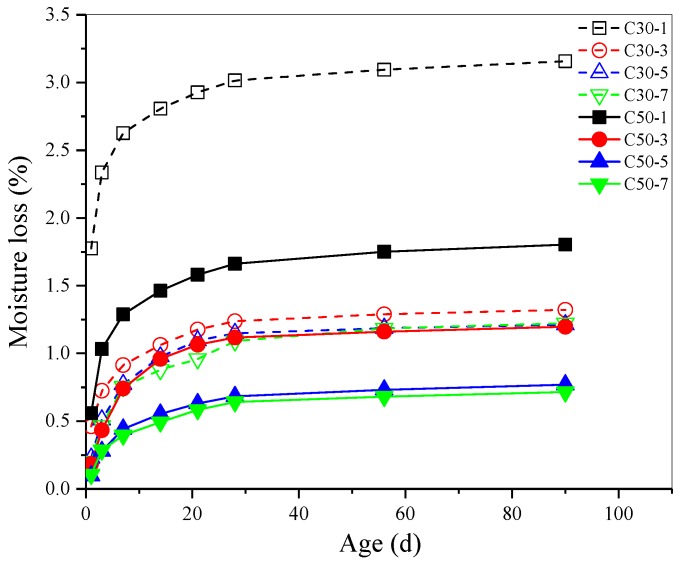
Effect of early age curing on moisture loss of AASC.

The effect of early age-curing methods on drying shrinkage and moisture loss of AASC is shown in Figure 3 and Figure 4 separately. Under the same curing conditions, drying shrinkage rate of C30 grade concrete was higher than that of C50 grade concrete at the same age. Because higher w/b of AASC resulted in more free water inside of concrete. After the specimens were placed in the drying shrinkage chamber, internal free water evaporated. So higher free water inside of concrete led to a greater shrinkage deformation [37]. The results in Figure 4 indicate that the moisture loss of C30 concrete at 3 d was more than 3 times that of C50 concrete at the same age, while the moisture loss at 90 d was almost 2 times.

Higher early curing temperature reduced drying shrinkage rate of AASC obviously. Drying shrinkage rate of concrete cured in water at 40 °C and 60 °C was much lower than that of concrete cured in water at 20 °C. When the water curing temperature increased from 20 °C to 60 °C, the drying shrinkage of C30 concrete at 90 d decreased from 1028 μm/m to 202 μm/m. C50 concrete also show similar trend, the drying shrinkage at 90 d decreased by 80% when the water curing temperature increased from 20 °C to 60 °C. The moisture loss was also highly dependent on the temperature of curing water. When the curing temperature increased from 20 °C to 60 °C, the moisture loss of C30 and C50 AASC decreased by 58% and 33% respectively. More hydration products formed under elevated temperature due to the acceleration of hydration process, meanwhile more water participated in the hydration of slag. So, the lower moisture loss should be attributed to the denser microstructure and less free water inside of AASC.

Comparing drying shrinkage of AASC cured in water and in air with a RH of 60% and 80%, it could be found that curing in water resulted in lower drying shrinkage. It should be noted that this phenomenon was more obvious for C30 AASC. Figure 4 show that the specimens cured in water lost much more water after being moved into the drying shrinkage chamber. Even though free water on the surface of specimens cured in water was wiped off after curing, much water filled in the open pores contributed to the higher moisture loss. For samples cured in air with a RH of 60% or 80%, some water evaporated during the curing period thus the moisture loss was lower in the drying shrinkage chamber.

In the case of CO_2_ curing, RH was important for drying shrinkage of both C30 and C50 AASC. Drying shrinkage of AASC decreased significantly with the increase of RH in the CO_2_ curing chamber. No matter the RH, CO_2_ curing led to much lower drying shrinkage compared to the samples cured in 20 °C water. Cured in CO_2_ chamber with a RH of 80% for 48 h, the drying shrinkage decreased by 49% and 53% respectively for C30 and C50 AASC. When cured in the same RH environment, CO_2_ curing did not show significant effect on moisture loss.

### 3.3. Products and Microstructure of AAS Pastes Cured in Different Conditions

**Figure 5:**
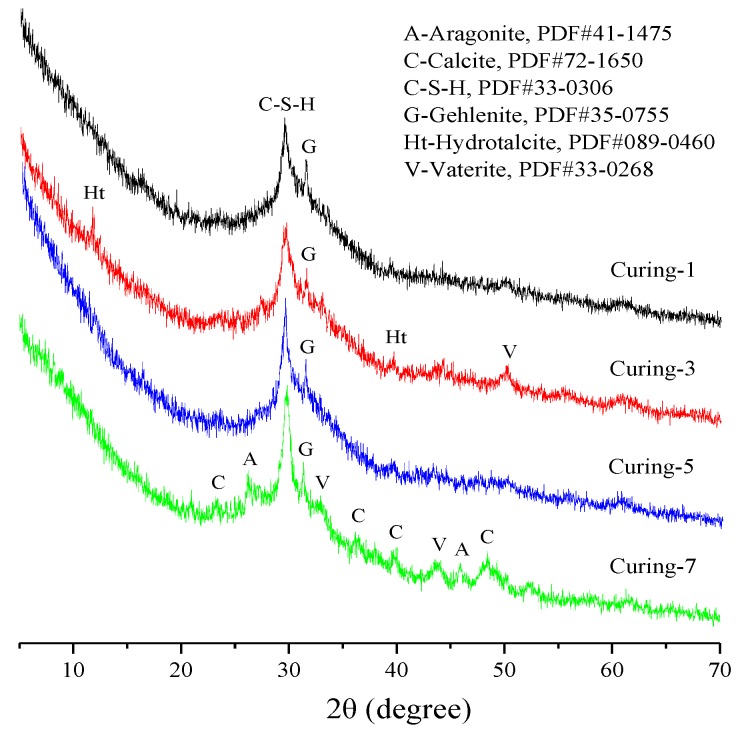
XRD analysis of AAS pastes.

**Figure 6:**
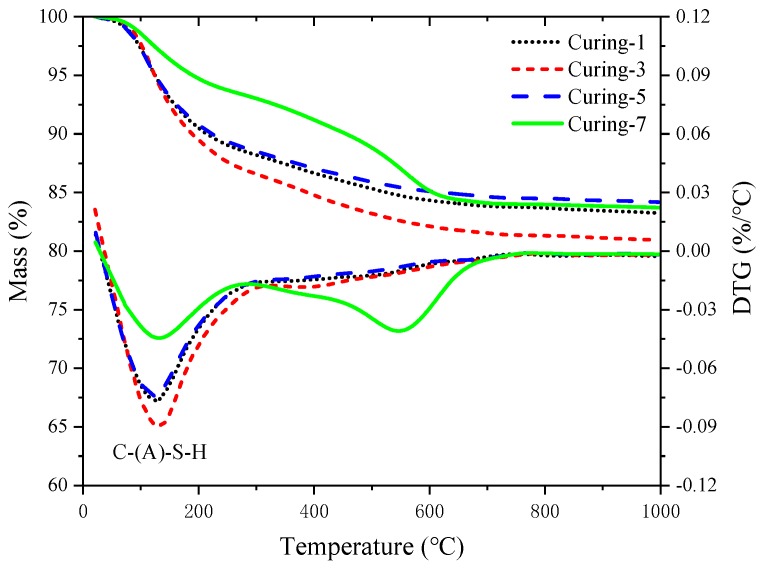
TG-DTG analysis of AAS pastes.

**Figure 7:**
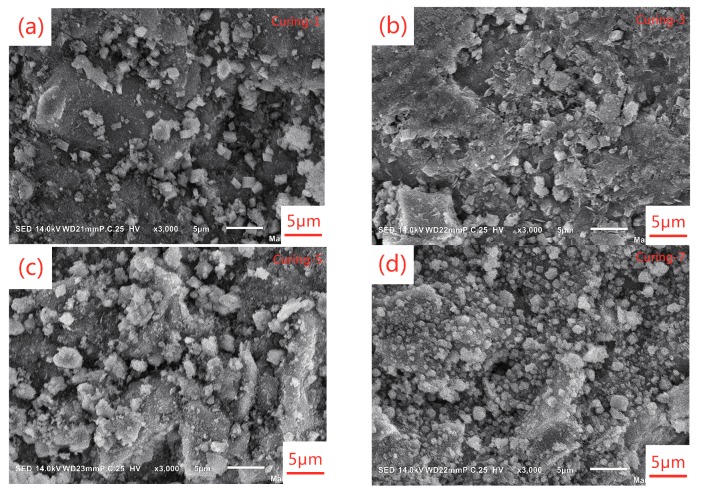
SEM images of AAS pastes: (**a**) 20 °C water; (**b**) 60 °C water; (**c**) air, 20 °C/60%RH; (**d**) CO_2_, 20 °C/60%RH.

**Figure 8:**
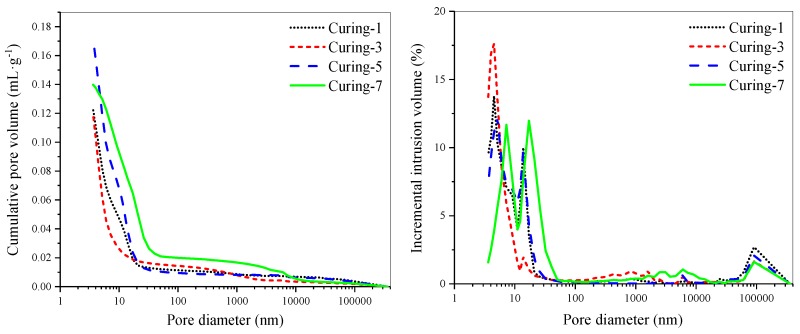
Effect of early curing on pore structure of AAS pastes.

AAS pastes with a w/b of 0.42 and alkali dosage of 5% were cast and demolded after 24 h, then cured in different conditions for 48 h. Finally, XRD, TG-DTG, SEM, and MIP were carried out to analyze the composition of hydration products and their microstructure. The results are presented in Figure 5, Figure 6, Figure 7 and Figure 8.

According to the XRD patterns in Figure 5, the wide dispersion ring around 2θ of 30° indicated that the main hydration product of AAS cement was C-(A)-S-H gel in an amorphous form. Also, minor amounts of gehlenite could be found at around 2θ of 32°, it demonstrated that slag hydrated incompletely due to the very short curing time. Peaks at 2θ of 12° and 40° indicated the formation of hydrotalcite in the sample cured in 60 °C water. For the sample cured in CO_2_ curing chamber, the broadness around 2θ of 30° decreased significantly and some new peaks associated with calcite, vaterite, and aragonite were found. It means that carbonation during the CO_2_ curing resulted in the decalcification of C-(A)-S-H and formation of calcium carbonate in the form of calcite, vaterite, and aragonite. The results were consistent with He et al. [38], Puertas et al. [39] and Bernal et al. [40], they found calcite, vaterite, and aragonite coexisted in carbonated AAS binders. While Li et al. [36] found that the carbonation products of AAS cement were mainly in the form of calcite and vaterite. The different results could be attributed to the different experimental conditions, including but not limited to, the age of samples, the CO_2_ concentration, and the exposure duration.

The TG-DTG test curves of the samples cured under different conditions are presented in Figure 6. A continuous weight decrease was observed within 20 °C to 950 °C, which was caused by the loss of adsorbed water, dehydration of products, and decomposition of calcium carbonate. Two endothermic peaks were noticed in the sample cured in CO_2_, while only one peak was found in the other samples cured in water or air. The weight loss in the range of 20 °C to 250 °C was mainly caused by the loss of water in the interlayer of C-(A)-S-H, so greater weight loss in this range corresponded to more hydration products. The curves in Figure 6 show that higher curing temperature led to more C-(A)-S-H formed and higher hydration degree. From the green curve in Figure 6, we find that carbonation occurred during CO_2_ curing period and resulted in less C-(A)-S-H formed. The characteristic peak between 450 °C and 700 °C indicated the decomposition of calcium carbonate formed during CO_2_ curing period [41]. The decomposition temperature in this study was lower compared to calcite, Šauman et al. [42] got similar results and attributed it to the imperfectly crystallized formation or finer crystal structure of carbonation products.

The microstructures of AAS pastes cured under different conditions are shown in Figure 7. Comparing Figure 7a,b, it could be found that more C-(A)-S-H gel formed at elevated temperature and resulted in denser microstructure. It can also explain the significantly higher compressive strength of samples cured under elevated temperature. The hard solid skeleton also led to lower drying shrinkage. Considering the effect of RH on microstructure of AAS pastes, insufficient water during the curing period resulted in loose microstructure as presented in Figure 7c. From Figure 7d, we can find that plenty of calcium carbonate formed under CO_2_ curing and filled in the pores, thus denser microstructure formed compared to the sample cured in air of the same temperature and RH (Figure 7c).

The effect of early age-curing methods on pore structure of AAS pastes was studied by MIP. The test results are shown in Table 8 and Figure 8. Curing in elevated-temperature water accelerated early hydration reaction of AAS pastes, and generated more C-(A)-S-H gel to make its structure denser. Therefore, the total porosity, average pore diameter, and median pore diameter of this specimen was the smallest of all groups.

Comparing the results of samples cured in water of 20 °C and air of 20 °C and 60% RH, it could be found that water curing reduced the total porosity from 28.78% to 23.01%, but the average pore diameter and median pore diameter was not affected much. In CO_2_ curing environment, hydration products of AAS pastes reacted with CO_2_. The generated calcium carbonate filled in the pores then led to the decrease of total porosity. Meanwhile, CO_2_ curing resulted in the increase of average pore diameter, because carbonation caused decalcification of the C-(A)-S-H gel and destroyed the microstructure. This finding is consistent with the results of Puertas et al. [39].

## 4. Discussion

Drying shrinkage of cementitious materials is mainly caused by evaporation of the water in pores into environment, the main mechanisms of drying shrinkage were summarized as surface energy, capillary stress and disjoining pressure [3,16]. During measurement of drying shrinkage, normally hydration of binders continues which results in autogenous shrinkage. Therefore, in all the cases autogenous shrinkage is included in the measurement results of drying shrinkage.

The results presented above show that curing in water of elevated temperature decreased drying shrinkage of AASC significantly. It could be attributed to several reasons. Firstly, the hard solid skeleton formed under high-temperature curing was beneficial for mitigating drying shrinkage. When cured at 60 °C, the compressive strength of AASC at 3 d reached and even exceeded that of AASC cured at 20 °C. Secondly, the decreased moisture loss could also help to decrease the drying shrinkage. The moisture loss of AASC cured in high-temperature water was reduced due to the formation of denser microstructure, which could be confirmed by the SEM images presented in Figure 7 and the porosity results presented in Table 8. Thirdly, the pore-size distribution of AAS pastes cured in heat water was good for decreasing drying shrinkage. Collins and Sanjayan [14] regarded higher total volume of mesopores ranging between 1.25 nm and 25 nm as the main reason for the higher magnitude of drying shrinkage of AASC. From Table 8, we can find that the proportion of pores smaller than 25 nm was also reduced slightly by heat water curing compared with samples cured in water of 20 °C. Finally, autogenous shrinkage of AAS pastes accelerated by elevated-temperature curing in the first few days was not included in the results of drying shrinkage because the measurement of drying shrinkage was started after curing. Cartwright et al. [18] studied the autogenous shrinkage of AAS mortars mixed with different alkaline activator. When the modulus of water glass was 1.22, water to binder ratio was 0.47, and the binder to sand ratio was 0.33, they found that the autogenous shrinkage accounted for around 60% of the drying shrinkage at 90 d. Heat water curing accelerated hydration of AASC, most of autogenous shrinkage occurred during the curing period before the initial length measurement and was not included in the results of drying shrinkage measurement. In the following period of drying shrinkage testing, autogenous shrinkage was quite low because the further hydration rate of AASC was extremely slow. This could contribute to the enormous reduction of drying shrinkage caused by heat water curing.

CO_2_ curing changed the total porosity and pore-size distribution of AAS pastes. According to the results listed in Table 8, it could be found that the total porosity and proportion of pores smaller than 25 nm was reduced by a large margin. As presented in Figure 7d, we can see that calcium carbonate formed by carbonation process filled in the pores of AAS pastes and led to the reduction of total porosity. While the median pore size increased from 7.2 nm to 14.3 nm, because decalcification of C-(A)-S-H coarsened the pores. The capillary pressure decreased with the increase of pore size, then it resulted in the decrease of drying shrinkage of AASC compared with that of AASC cured in air with the same temperature and RH.

**Figure 9:**
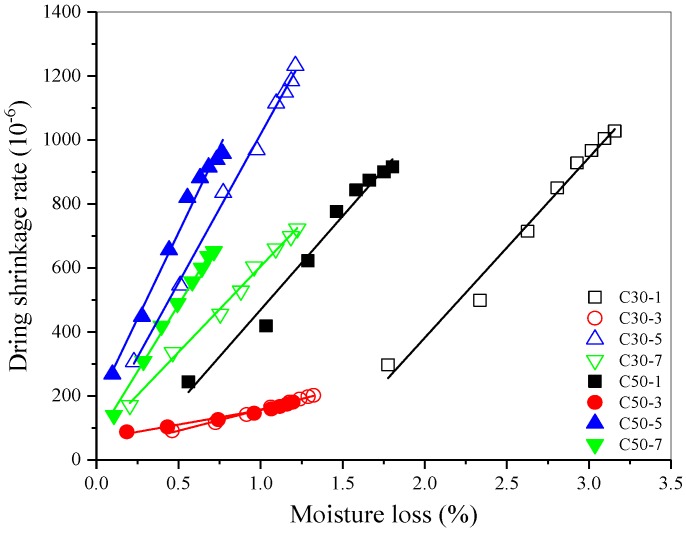
Correlation between drying shrinkage rate and moisture loss of AASC.

Figure 9 is a diagram showing the correlation between drying shrinkage ratio and moisture loss of AASC. There is a good linear relationship between drying shrinkage rate and moisture loss for an AASC cured in the same environment. All the correlation coefficients were greater than 0.97762 (as shown in Table 9). The slope of C50 AASC was greater than that of the C30 AASC cured under the same condition. The pore size of C50 concrete matrix was finer than that of C30 because of the lower w/b. When the moisture evaporated from finer pores, it produced larger shrinkage stress and caused greater shrinkage [15,43,44]. The results are consistent with that of Thomas et al. [16], who found that increased sensitivity suggested a finer porosity, while decreased sensitivity suggested a coarser porosity.

For the same grade AASC, the higher early curing temperature, the smaller the slope of linear fitting line of drying shrinkage ratio and moisture loss. Heat water curing accelerated hydration and formed a harder solid skeleton, which had a higher resistance to capillary stress. Then the drying shrinkage sensitivity of AASC to water loss was reduced obviously. Water curing decreased the slope of linear fitting line by a large margin, it indicates that the sensitivity of drying shrinkage to moisture loss was reduced significantly. The main reason could be attributed to the reduction of total porosity as discussed previously. Reduction of the total porosity and coarsening of pore-size distribution induced by CO_2_ curing led to an obvious decrease of the slope of linear fitting line between drying shrinkage ratio and moisture loss.

## 5. Conclusions

In this paper, the effect of early age-curing methods, including curing in water of different temperature, air curing and CO_2_ curing, on drying shrinkage, moisture loss, and strength of AASC was studied. Combined with microscopic test such as SEM, MIP, XRD, and TG-DTG, conclusions could be drawn as follows:

1. Under the same curing condition, C50 AASC showed lower drying shrinkage but higher drying shrinkage sensitivity to moisture loss than C30 concrete. This result was due to the lower total porosity and finer pore size of C50 AASC caused by the lower w/b.
2. Water curing decreased drying shrinkage of both C30 and C50 AASC compared to air curing, while it was more effective for C30 AASC. Moisture loss was increased by water loss; however, the decline in total porosity and proportion of pores smaller than 25 nm led to the decrease of drying shrinkage.
3. Curing in water of elevated temperature resulted in a significant decrease of drying shrinkage for both AASC. Increasing water temperature from 20 °C to 60 °C, the drying shrinkage of both AASC declined by 80%. Under high-temperature curing, most autogenous shrinkage of AASC occurred in the first few days because hydration was accelerated. After measurement of drying shrinkage was started, recorded autogenous shrinkage of AASC cured in elevated-temperature water should be much less than that of AASC cured at normal temperature. It is one of the most important reasons for the reduction of drying shrinkage. Furthermore, the increase of compressive strength, reduction in total porosity, and proportion of pores smaller than 25 nm could also contribute to the fall of drying shrinkage.
4. CO_2_ curing was also an effective way to reduce the drying shrinkage of AASC. In the present study, the drying shrinkage of C30 and C50 AASC decreased by 49% and 53% respectively. The products of carbonation occurred in the curing period were calcite coexisted with vaterite and aragonite. Decalcification of C-(A)-S-H coarsened the pore-size distribution and decreased the total porosity, thus drying shrinkage was declined by a large margin.

## Figures and Tables

**Table 1:** Chemical compositions of GGBFS (wt.%).

SiO_2_	Fe_2_O_3_	Al_2_O_3_	TiO_2_	CaO	MgO	MnO	SO_3_	K_2_O	Na_2_O	Loss
32.70	0.50	14.03	2.13	39.00	8.99	0.40	0.20	0.41	0.25	0.78

**Table 2:** Physical properties and chemical compositions of water glass.

W_(SiO2)_ (%)	W_(Na2O)_ (%)	W_(water)_ (%)	Baume Degree (°Bé)	Density (g/cm^3^)	Modulus
28.13	11.09	49.72	44	1.452	2.62

**Table 3:** The basic performance of manufactured sand.

Apparent Density (kg/m^3^)	Bulk Density (kg/m^3^)		Void Ratio (%)		Powder Content (%)	Fineness Modulus
	Loose	Tight	Loose	Tight		
2680	1300	1540	51.0	43.0	5.8	2.9

**Table 4:** Chemical compositions of crushed limestone (wt.%).

SiO_2_	CaO	Al_2_O_3_	Fe_2_O_3_	SO_3_	MgO	Na_2_O	Loss on Ignition
2.50	54.03	0.60	0.36	0.01	0.54	0.08	36.60

**Table 5:** The basic performance of crushed limestone.

Particle Sizes (mm)	Apparent Density (kg/m^3^)	Bulk Density (kg/m^3^)		Void Ratio (%)		Clay Content (%)
		Loose	Tight	Loose	Tight	
5-10	2670	1380	1470	48.3	44.9	0.7
10-20	2670	1400	1520	47.6	43.1	0.5

**Table 6:** Mixture proportion of AASC (kg/m^3^).

Group	w/b	Slag	Sand	Gravel	Water	Water Glass	NaOH
C30	0.50	400.0	734.8	1102.2	148.6	103.4	11.0
C50	0.42	400.0	785.0	1084.0	116.6	103.4	11.0

**Table 7:** The early age-curing methods.

Group	Temperature (°C)	RH (%)	Time (h)	Curing Conditions
1	20	100	48	Water curing
2	40	100	48	Water curing
3	60	100	48	Water curing
4	20	80	48	Air curing
5	20	60	48	Air curing
6	20	80	48	CO_2_ curing
7	20	60	48	CO_2_ curing

**Table 8:** Effect of early age curing on pore structure of AAS pastes.

Group	Total Porosity (%)	Average Pore Diameter (nm)	Median Pore Diameter (nm)	Pore-Size Distribution (%)		
				<25 nm	25–5000 nm	>5000 nm
1	23.01	7.2	7.0	88.68	5.30	6.02
3	18.63	6.1	5.2	85.72	10.69	3.59
5	28.78	7.3	7.2	92.10	3.34	4.56
7	21.31	12.2	14.3	73.42	18.95	7.63

**Table 9:** Correlation analysis results between drying shrinkage rate and moisture loss of AASC.

Group	C30-1	C30-3	C30-5	C30-7	C50-1	C50-3	C50-5	C50-7
Slope	563.35660 ± 30.33916	132.56786 ± 4.36318	929.33223 ± 20.83994	536.21887 ± 15.43849	585.73686 ± 35.86805	90.89841 ± 5.61508	1075.27965 ± 53.27048	835.91454 ± 22.87730
R^2^	0.98290	0.99354	0.99699	0.99505	0.97800	0.97762	0.98549	0.99553
